# Of What Does the Universe Consist?

**Published:** 1914-03

**Authors:** Roswell Park

**Affiliations:** Professor of Surgery, 1883-1914


### BUFFALO MEDICAL JOURNAL

VOLUME 69 MARCH, 1914 No. 8

### ORIGINAL ARTICLES

### Of What Does the Universe Consist?

BY ROSWELL PARK, A.M., M.D., LL.D.
Professor of Surgery, 1883-1914

Lecture at the University of Buffalo, February 1, 1914.*

THIS question has baffled philosophers of the past and present and will baffle those of the future, in all probability, until some new method of bringing information to our finite senses is afforded from some most unexpected source.

But suffice it for oui present purposes to say that the general concept of the universe implies an inconceivable, limitless space, occupied by something to which, for want of a better name, we give the term—*matter*. To define *space* is as impossible as to define matter. Aided by what knowledge we have we permit our imaginations to run riot, and then try to conceive of space conditioned by dimensions to which we can attach no limit, and then, beginning again where that concept leaves us, we may endeavor to enlarge its boundaries by some similar process, and even then feel that we have not reached the limit of that which is our limit, if limit there be.

So, too, regarding *matter*. The usual concept of matter is as of something which occupies, possibly fills, this space. Such appreciation of matter as we really have must come to us through the senses of sight, hearing, smell, taste and touch, which reduce it to very meagre terms. Applying processes of reasoning to facts accumulated through the senses we gather a certain amount of knowledge regarding distant objects, such as the planets, the sun and the stars. From these distant bodies, by means of some otherwise inappreciable medium, there come to us *vibrations,* which we interpret as material expressions of objects far distant from us.

This knowledge is then transmitted apparently by means of vibrations, and it is impossible to conceive of vibrations without supposing a medium which may vibrate. This medium has, in time past, been called the *ether* of space—and, while inseparable from our concept of matter, has nevertheless taxed the imagination of scientists to the very utmost. Until very recently this

* This is the last paper written by Dr. Park and, so far as known, the only one not already published or awaiting publication at the time of his death.

ether of space has been regarded as a subtle, imperceptible, wonderfully tenuous medium, more rarefied than any known gas, capable of penetrating and permeating every other substance known, and differing from every other substance by virtue of this very tenuousness and permeability. To its vibrations were ascribed the phenomena of light, just as those of sound are to be ascribed to vibrations of the air. Only through this ether also could be transmitted that most mysterious agency known, namely, *gravitation.* For all the known phenomena of light certain time is required, and the rapidity of its transmission has been accurately recorded by methods and instruments of extreme precision. For the phenomena of gravitation no satisfactory explanation has ever been afforded. Our concept of it is as of a mysterious force which makes itself felt as much more rapidly than light as light does than sound, perhaps even exceeding this celerity. Nothing that is known or could even be postulated regarding gravitation would meet a single actual need called for in its explanation. Nevertheless, that a force or an agency could be exerted through nothing, i. e., absolutely *no medium,* is to negative any concept that can be entertained.

So rapid has been the progress of accumulation of data, and of the application of rigid mathematical formulation and calculation to their interpretation, that I, for instance, see myself today in a position where I have been compelled to forget nearly all the elements of science which as a boy I acquired; that is to *unload them* and to take on an added burden of facts and of logical and rigorous analysis which, for one placed like myself, is doubly hard, because of the double task imposed; nemely, to *unlearn* the *old* and to *acquire* the *new.*

It is in regard to some of these facts acquired in this effort that I hope to interest you today.

More than two thousand years ago the ancient Greek philosophers taught that the universe consisted of four elements—earth, air, fire and water. This teaching was long ago abandoned, and the world was taught that the universe is made up of certain so-called "elements," meaning thereby varieties or forms of matter, each of which has been reduced to its simplest, utmost divisible and ultimate form, and that from this form no further reduction from anything else was possible. As time went on, and this time has mainly been included with the past two centuries, these elementary substances grew in number, from one containing but few units, up to perhaps fifty, when I was a lad and first studying, and now to about ninety with, possibly, others to follow, as I shall endeavor to make appear to you. A few of these, like gold, silver, etc., found in their native state, were known to the ancients; others have been discovered by accident, a considerable number of them by aid of the spectroscope, and a few of them by a refinement of chemical manipulation and laboratory jugglery, which must for all time challenge the admiration of the scientific world. As the accomplishment of an actual marvel in this last respect I refer to the isolation and study of the radium elimination, by Rutherford, who has thereby not only added the element—Niton—to the list, but has demonstrated a skill in manipulation of minute amounts that should characterize him as the veritable juggler of the scientific world.

It seems but yesterday that we were compelled to say that space is occupied by matter, by force and by ether. So rapid has been the march of reason and science that today the scientific world is asking whether force and electricity do not constitute matter, and whether, therefore, space is not *filled by force and ether?*

Here must come in the legitimate use of the reasoning faculty to which Tyndall called attention in one of his charming essays, speaking of it as the "Scientific Use of the Imagination." To it most of our progress has been due; without it further advance would be impossible. It includes that *tour de force* of the mind which first postulates a certain necessity and then leads on and upward to its demonstration. For instance, the concept of the atom is as of something so small that a knowledge of itself can never come directly to our senses, and we know it only by studying its effects, much as one may regard the flash of lightning as a manifestation of some unseen or unknown force. Many centuries ago its existence was postulated by Leucippus, by Lucretius and by Democritus. The very term—*atom*—means the ultimate, the indivisible, last particle of matter into which it can be regarded as possibly divided. Existence of the atom has been demonstrated during recent years to the satisfaction of all capable of appreciating it. Then came a demand for something smaller or finer yet, and thus were postulated the corpuscles, the ions, and the electrons. About these terms some confusion exists, the terms being variously applied by various authors.

These terms do not comprise the concept to which we give the name—*molecule*—implying thereby the union of several atoms, or even of a considerable number of them, which act as a unit and apparently comprise the last stage in the subdivision of a given substance. In other words the molecule of a substance, if reduced to atoms would simply contain its component parts.

As discoveries have accumulated the relations which ever exist between mathematics and physics, including chemistry, become more evident and more complicated. The ideal chemist, the electrician, or the physicist of today must be an expert mathematician. Consideration of these relations takes us back even to the old days of Pythagoras, who laid down the maxim that the "universe is founded on number." The necessity for this relation may perhaps be illustrated by the fact that the so-called "conic sections," namely the circle, the ellipse, the hyperbola and the parabola, were all known to the ancient Greek geometers, but by them were regarded as of purely speculative value. Notwithstanding it was these which enabled Kepler, some twenty centuries later, to announce with precision the motions of the planets about the sun; while, later yet, most of our astronomers have shown how intimately concerned are conic sections and astronomy. Again it was the theory of "Imaginary Exponentials" applied to the study of vibrations that made wireless telegraphy possible; and it was the "Theory of Groups" which enabled our own American scientist, Michelson, to develop by experiment the existence of that feature of *"Relativity"* which has been such an immense advantage to the investigations of science. Therefore, it has been mathematical theories which have made possible the evolution of the theory and the practise of physics, as we now have them, in so many various manifestations.

Probably the only opportunity in this lecture, today, of illustrating the relation of number to nature applies in the so-called *"Periodic Law,"* and it is largely to this that your attention is invited; and it is solely for its presentation that I constructed the accompanying charts.

But first something about the atoms and the Atomic Theory.

A few points in the history of the atomic theory may help to better illustrate our present views regarding them.

In 1789 Sir Humphrey Davy and Count Rumford (who, by the way was an intimate friend of one of my own forbears) upset the theory of heat held at that time, and showed that it was really a mode of molecular motion, any given substance being hot by virtue of intensified molecular activity within it, and the reverse down to absolute zero.

A year or two later Young upset the corpuscular theory of light, at least for the century following, and apparently proved that it was really a wave motion in that newly conceived medium the ether.

Then, in 1800, these men, along with Volta, who discovered the Electric Pile, founded the *Royal Institution,* which has really done more, in the decades following, than any other body of learned men to advance the science of physics.

In 1802 the dark lines in the Solar Spectrum were first noticed by Wollaston, who in this way laid the foundation for the science of spectrum analysis, to whose elaboration we are probably as much indebted as to any other department of science.

In 1803 (the year following) Dalton advanced his Atomic Theory, and thus gave the impetus to that vast advance in chemistry and, later, to chemical physics, which has permitted this statement, e. g., of the views of today.

We will pass over the intervening years of the past century, and turn to its concluding decades, and speak briefly of the discoveries of Crookes, who, in 1878, produced his *Cathode Rays,* and claimed that they demonstrated a *new, fourth* or *radiant state of matter.* Then Hertz discovered that these rays might traverse this sheets of metal, and, in 1893, Lenard brought them outside of the vacuum tube into the air.

It was in 1895 that Roentgen developed Lenard's work and discovered, as it were by accident, the so-called *X-Rays,* quickly demonstrating their chief properties. The distinction should be made here, as well as later if they are again considered, that the X-Rays should not be confused with the Cathode Rays—the latter are given off only in a highly exhausted vacuum tube. The X-Rays are not produced until the Cathode Rays undergo some sollosion as, for example, with the glass wall of the tube, in consequence of which collision they assume the properties and peculiarities now generally ascribed to them.

Next year, 1896, quickly followed the discovery of radio-activity, by Becquerel, in Uranium and ores containing it.

The following year, 1897, J. J. Thomson demonstrated that the Cathode Rays are streams of actual matter, and consist of particles no larger at least than one one-thousandth of the size of the hydrogen atom, which, up to that time, was the smallest particle of matter known to scientists. Of these we shall have more to say.

In 1903 Radium was discovered by the Curies. During the three or four years following, by the efforts especially of Rutherford and Soddy, there was developed a new science of sub-atomic chemistry, in connection with the disintegration theory of radio-activity, which has astonished not only general students of the subject but expert scientists. In fact in the development of the feature of radioactivity, Rutherford and his pupils and assistants have astonished the world by their marvelous grasp of scientific principles and their marvelous ingenuity in devising demonstrations, as shall later appear.

The past century also has contributed wonderfully to the elucidation of material phenomena, beginning with Oerstedt, who, in 1821, first showed the intimate relation between electricity and magnetism; then Faraday, who, in 1831, developed the Laws of Induction, and, later, the relations between electricity, magnetism and light, which were subsequently and collectively developed by Maxwell in his Electro-Magnetic Theory of Light, whose final verification came through the experimental researches of Hertz, to whom we are really indebted for wireless telegraphy.

The introduction of the induction coil, permitting the production of high tension currents, especially through highly exhausted tubes, gave the first impetus to the minute study of what goes on within that vacuum through which is passing an electric current. Crookes (now Sir William Crookes), to whom we are in the main indebted at least for our introduction to this subject, showed that the rays which proceed from the cathode, i. e., the negative pole, through a vacuum, actually consist of streams of minute particles, traveling at intensely high speed, each of which carries a *negative* charge of electricity. In this way we account for their diversion, and the fact that such a stream widens in its passage, and constitutes that fourth state of matter which is neither solid, nor liquid, nor gaseous, to which he gave the name of *Radiant Matter.* Over this state there raged a tedious controversy for two years. It seems now, however, to be well established that this is in effect an actual stream of particles of matter whose actual mass cannot be considered as greater than that just mentioned. That matter may exist in this minute form, in fact *must* so exist, has been shown in so many different ways that it may now be accepted as indubitable and authentic. The most demonstrative experiment consists in diverting this stream by a magnetic current; unless it were an actual stream of particles it could not be so diverted.

The result of these studies has been to make it plain beyond all possibility of doubt that *electricity, like matter, is atomic in structure;* that there is a so-called unit charge of electricity; that all other charges are multiples of this; that these particles may be given off from the white hot filament of an incandescent lamp, or from zinc exposed to ultra violet rays; by passage of X-Rays through a gas, and by radiation from a radioactive substance. In fact the charges associated with this discharge have been measured, and have been so compared and contrasted with each other as to make positive their existence and identity.

In 1898 Thomson made one of the most beautiful experiments ever devised, based on the formation of clouds. In this he showed that out of the small particles which constitute an electric charge it was possible to produce a cloud of watery vapor, which could be charged in accordance with the requirements of the conditions, especially that of the rarefaction of the air contained within the vacuum tube. As the result of experiments similar to these conducted by many observers, who have taken into consideration all possible points, the conviction has been forced that there exists an ultimate charge or an actual atom of electricity, and that when we deal with the cathode ray we have to deal with an actual entity, not larger than one one-thousandth of the smallest particle (the hydrogen atom) ever previously conceived of and probably much smaller. These ultimate particles seem to be identical, no matter from what gas or source derived; hence, apparently they are the ultimate constituent of matter. It is interesting to contrast the inevitable deductions from these studies with "Prout's hypothesis." Prout believed the atoms of all other elements to be built up of hydrogen atoms, simply because these were the smallest recognized. According to this still later view it might appear, and to some does so appear, that all atoms, even those of hydrogen, are at least to some extent, built up from particles of one common but individual kind.

With this view it would seem necessary to give some identifying term or name to these new particles. By some they have been spoken of as corpuscles. Today they are most generally and acceptably designated as *Electrons*. If, then, one were asked—"What constitutes an electron?"—he should reply that it is an infinitesimal particle, whose apparent or calculated mass or volume is about one one-thousandth that previously ascribed to the hydrogen atom, and that it bears a negative charge of electricity, equal to the number $3.4x10—^{10}$, i.e., 1/3.40,000,000,000 mm.

We have no time more than to mention the so-called Canal Rays described in 1896, by Goldstein, whose stream can be deflected by both magnetic and electric fields, although more feebly than the Cathode Rays, which would indicate that these particular rays must consist of particles larger than that above mentioned, each of which carries a positive charge instead of a negative.

J. J. Thomson has just concluded some most remarkable researches regarding the nature of these particles which are thus projected through a small opening *behind* the cathode instead of from in front of it. Their importance obtains especially in this fact, that by his researches there has been opened up a new method of analysis, more sensitive even than that afforded by the spectroscope, since with only one-hundredth of a milligram of a gaseous element present in the tube, and an exposure less than one-millionth of a second, its atomic weight can be determined within an accuracy of one per cent., and not alone this but even its physical condition. Scarcely any discovery in the whole list of recent developments is more interesting or important than this, and yet we hear but little of it.

When dealing with Radium we shall have, again, to speak of the X-Rays, since the so-called Gamma Rays are those from which the public seem to expect much in the way of therapeutic advance and the cure of various diseases. It is most important then to know what the X-Rays are. Here again Thomson has figured brilliantly in the demonstration that the X-Rays are really due to pulses in the ether, produced by sudden stoppage of electrons in their career; which means that from the negative charge of an electron a Faraday ray of force, traveling with it through the ether with the same speed, which is perhaps one-third of the speed of light, is suddenly brought to a stop by a collision with some other particle. The result of this is a new impulse, which, being single, that is, not a train of waves, can be made to show the ordinary phenomena of reflection, refraction, and the like, which only a wave can produce. It is, nevertheless, an instantaneous and electro-magnetic disturbance in the ether. This is why the X-Rays originate at the point of impact or collision of the Cathode Ray, and why they are not subject to any influence by a magnetic field. It means that *one* Cathode Ray, colliding with *one* atom, may produce one *X-Ray,* and that this X-Ray colliding with another atom may set up again a Cathode Ray; this process going alternately, but gradually weakening until it ceases. That the X-Rays are a corpuscular stream furnishes the explanation of their extreme penetrating power. This must be ascribed to the fact that they are electrionic in size, that is far smaller than the atom, and that they are electrically neuter, i. e., negative.

### The Periodic Law.

Up to 1860 the proportions according to which elements unite had been regarded as of little importance, and no attempt had been made to study or to take advantage of them, nor to calculate them with absolute accuracy. For instance, until that time three different weights had been proposed for carbon. But this situation became intolerable, and in 1860 an International Conference took place, at which Cannizzaro read an epoch-making paper in which he called attention to their real significance. Attention had been already called to the fact that iron, nickel and cobalt had almost the same atomic weight, and to the fact that the weights of chlorine, bromine and iodine seemed to increase by a fairly constant increment. The English chemist, Nowlands, took the first definite step, when he showed that by arranging the elements in the order of their atomic weights it would be found that every eighth element was a sort of *replica* of the eighth preceeding. These "eighths" he called the *octaves.*

In 1869 the Russian Mendeleeff, and the German Lothar Meyer, independently attacked this matter, the former giving it by far the more comprehensive study. Thus it came about that Mendeleeff put in words the so-called Periodic Law, in which he stated that "*all of the properties of the elements, both chemical*

*and physical, vary in a periodic fashion in accordance with their atomic weights."* With amendments and additions the chart placed before you represents the latest acceptance of Mendeleeff's views.

In this they are arranged vertically in groups and horizontally in series.

The following are among the most notable features that may be briefly pointed out in this table:

Follow each series horizontally from groups one to seven, and there will be noted a constant increase in their atomic weight, differences between them being nearly the same.

Note that the eighth element, namely, Sodium, resembles the first, Lithium, beneath which it is placed.

This resemblance holds good especially for the first four rows.

When we come to series or row five, there appears quite a change in this respect, and resemblances are greater between alternate series, thus the sixth resembles the fourth, the seventh the fifth, the eighth the sixth, and the tenth the eighth (as indicated in the table).

Reading downward again, the various groups contain elements which are closely related in their properties. The resemblance is even more marked when alternate rows are compared.

Again, as the atomic weight increases common or characteristic properties become either much more distinctly or much less distinctly marked.

Again, reading downward, the elements tend to become more and more base-forming, and more metallic as the atomic weight increases. So again with their malleability, their melting point, and their specific gravity, these variations recurring with striking regularity.

The so-called *Triads* to which Döbereiner first called attention so long ago as 1829, meaning groups of three, and already alluded to, are placed in group eight in place of the single element they are supposed to represent.

Another striking feature is that read horizontally or in series the specific gravity is highest in the middle groups of each series.

A still more striking feature, which I could not present in these tables, is the so-called *atomic volume,* which is calculated by *dividing the atomic weight by the specific gravity.* It has been found that even more startlingly regular relations are found in these figures.

Again, read from left to right along the series and the elements become in turn more and more electro-negative. On the other hand, read downward in groups, from series two, they become more and more electro-positive.

One may well stop here and try to sum up the purpose and the value of this arrangement, as shown on this condensed table. The periodic classification has, at least, four practical values in that it permits us

(1) To classify elements in natural groups.

(2) To fix the atomic weights of elements whose equivalents are yet uncertain.

(3) To predict the possible existence and properties of elements not yet discovered.

(4) To correct errors in weights already assigned.

These are matters of far greater importance than at first were readily appreciated.

For example, the atomic weight of Indium could not be definitely determined until its valence was known. Experimental determination of this permitted its correct assignment in the table.

The fourth application has proved also of the greatest value. Mendeleeff held that many atomic weights had been incorrectly determined, and in almost every case his assumption was proven correct.

This table has, moreover, strikingly illustrated the value of "scientific use of the imagination," as, for instance, when Mendeleeff predicted, in 1871, that there would be discovered an element whose atomic weight would be about 69, of low melting point, of specific gravity 5.9, that it would decompose steam at red heat, that it would be more easily voltalized then aluminum and that it would be discovered by the spectroscope. Only four years later this prophesy, with further details, was strikingly verified, in the discovery of the rare metal known as Gallium, which was first detected by the spectroscope and proved to have properties practically identical with those which he had prophesied for it. Again, in the discovery of the so-called rare gases of the atmosphere. Just as soon as Argon had been identified, and Helium measured, and its A. W. determined, it followed naturally that the discovery of other gaseous elements would be announced—and these we have, their formulæ, etc., being given in the table.

True it is that there are yet not only vacancies but discrepancies in this table. Time fails in which to call attention to them, although a few of them are most glaring. It is not improbable that two or three at least, if not more, of the so-called elements are yet compounds whose primaries have yet to become known to us. This is the case, for instance, with Tellurium, which seems out of place in its present position. It is true also of Argon, with its atomic weight greater than that of potassium. These discrepancies, however, serve only to give added interest and to excite to still more accurate study, but there can be no doubt but that behind it all there is concealed some vast so-called Law, that is some statement which covers the nature and inevitable sequence of events in the constitution of matter, and the changes that go on within it.

I have constructed a separate table which I venture to call the *"Apocrypha,"* because it is not yet fully authenticated nor made "canonical" by the chemists. It contains the names of a number of elements discovered recently, most of them among the so-called rare earths. Seen by the spectroscope they appear to be elemental, but they have not been collected in sufficient amounts to assign them to any particular place in the general table, nor is it essential for our purposes they should be. Three of them—Actinium, Ionium and Polonium, will come in for further consideration when we deal with Radioactivity and Radium.

This consideration of the Periodic Law, based as it is upon atomic weight, has been deliberately brought under revision today for a double purpose. It is in and of itself a most fascinating introduction to the general study of chemico-physics, because it deals so intimately with the constitution of matter, and especially with the individual features attaching to each atom. In both these respects it forms a necessary introduction to the study of *Radioactivity,* if that is to be intelligently attacked. Unless one realizes that the atom is not to be regarded as formerly viewed, but is held to be a small cosmos within whose inconceivably minute boundaries there is constantly going on a perfect whirlwind of motion or activity, on the part of electrons, corpuscles, or call them what one will,—unless, I say, one can form some concept of something of this kind one can have no conception of what it means to have particles given off from a given atom by which its atomic wieght is reduced, its physical properties altered, and all this accompanied by certain phenomena of which the ingenuity of the chemist has enabled us to become aware; as when, for instance, from the atom of radium a particle flies off and strikes upon a zinc sulphide screen the fact becomes known because of the tiny flash of light which makes the impact, and then and there result one atom of Helium and one atom of Nitron, formerly known as Radium emanation; the atomic weight of the former being four, of the latter 222, and the total 226, representing the weight of an atom of Radium. Disintegration of the atom, then, is inconceivable without that modern and enlarged view of the atom itself which is now everywhere accepted, but which is a very different notion from that inculcated in me, when I was a boy studying elementary chemistry.

The charts herewith exhibited include first a graphic presentation of the facts underlying the so-called periodic law, as improved by Mendeleeff, who took advantage of what had been already done, who pursued his studies independently of Lothar Mayer, and who arranged his table in more illustrative and convincing form.

The other charts are enlargements, which contain in their first column the series as presented in Chart 1; then the name of the element with its symbol. In the next column is presented the valence of each element; this means its combining capacity. This combining capacity must have a standard, and for this purpose we select, as for so many, either the hydrogen or the oxygen atom. In the lettering at the left upper corner the letter "R" stands for the element, which ever it may be, while the figures attached to it represent the number of its atoms which combine with one atom of oxygen to form a molecule of the new substance thus made; or with hydrogen, since the relationship is preserved throughout each group.

In the molecule of water, for instance, whose chemical formula is H2O, we say that oxygen shows a valence of two, inasmuch as in this formation two atoms of hydrogen are required for each one of oxygen. In other words the valence of a given element is the power of the single atom to hold from one up to five, or even seven atoms of hydrogen.

There is no time now to explain how this atomic weight is calculated; in fact so difficult are some of these calculations that the exact weight beyond the decimal point is in a few instances still under discussion; nevertheless, it is known accurately enough for all our present purpose.

In the next column is given the specific gravity of each element. This means its ordinary weight as compared with water. Then follows the melting point of these elements not already fluid, or gaseous, or it gives the solidifying or the liquefying point if already condensed. These temperatures are given in both Centigrade and in Fahrenheit. Finally, on each table follows the date of the discovery of each element, with the name of the discoverer and its source.

For convenience in demonstration I have indicated by a marking in red each of those rare elements which are either rarely seen or are peculiarily difficult to procure, but which I have here on exhibition before you.

One of the most important features of this table are the figures in the column of *Atomic Weight*, given in red, which indicate the multiples of the first element in each group, with their steady increment which constitutes one of the most striking features of such tables. Certain gaps or vacancies will be noticed, and from what we know of the past the future will permit the discovery of elements to fill these vacancies. It was so, for instance, recently in the case of Radium, and one of the most excellent demonstrations of the value of this tabulation was afforded by Mendeleeff, who not only foretold the early discovery of at least three of the elements now included in this list, but had predicted with an extraordinary degree of exactness their physical characteristics and the combinations into which they would readily enter. (See above). No more striking verifications of scientific prophesy can ever be afforded than these predictions of Mendeleeff, and it is probably not expecting too much to believe that ere long there will be found elements to occupy every vacant space in this table. In fact, on the other (the "Apocrypha") chart included in this presentation, I have placed the names of a number of newly discovered elements, about most of which very little has yet been learned. Of at least three of these—Actinium, Ionium and Polonium, I shall have to speak when considering Radium. It will be seen that the others all have very high atomic weights, yet not enough has been learned about them to make one feel sure enough to insert their names in the large table.

### THE ATOM.

The present day view of the atom makes of it a deliberately balanced structure of greatest complexity, while the phenomena of radioactivity suggest that its direct balance is occasionally upset, whereupon it disintegrates with explosive violence. How can such prodigious energy be stored up within an atom? To come down to figures, both Einstein and Lewis conclude that the energy contained in a gram of matter (we might say, the energy constituting that gram of matter) is no less than 900 trillion ergs (or $9x10^{20}$ ergs), that figure being the square of the velocity of light in centimeters per second. If this figure is correct it means that an ounce of matter—any sort of matter—contains enough energy to blow nearly a million tons right off the earth.

How such prodigious energy is stored within the atom is still a mystery. But the phenomena of radioactivity have familiarized us with an astonishing reserve of energy contained within the atom, and absolutely unsuspected a few years ago. They have also brought home to us the suggestive fact that the mass of an atom may, in some rare cases, undergo a gradual diminution, accompanied by a relatively enormous evolution of energy.

The mathematical as well as the experimental methods of counting the atoms or the molecules, and of estimating their relative size, is another of the most fascinating yet difficult problems of modern science; nevertheless, it has all been done, and done with such relative accuracy, in so many different ways, and by so many different individuals, that checking up accounts as it were, or by comparison of results, there have been obtained estimates of whose accuracy no reasonable person can longer be in doubt. Even thirty years ago Professor Rowland of Baltimore, considering the spectrum of iron, with its thousands of lines, each indicating a different rate of vibrations, uttered the remark that, *"compared with an atom of iron a grand piano must be a very simple structure."* Of an aggregation of smaller particles the atom must then be built up. The electron has been shown to be of the order of 10-13 centimeters (one hundred trillionth), and the atom to have a diameter of $10^{-8}$ centimeters; in other words, one is the one-hundred thousandth of the diameter of the other, while the spaces between the electrons have a length probably of one hundred million times their diameter. This is in effect a sort of planetary system within the atom.

The velocity with which an atom of Helium is projected from Radium, as the latter is successively reduced to its lower forms, would require an electric field of more than four million volts, were it not that this energy is reduced by the internal conditions of its rotation within the radium atom, since a single electron moving in a single orbit must soon lose its energy, although if many electrons are working in the same orbit this is not true to the same degree.

J. J. Thomson has constructed a most interesting model of an atom, showing how only a certain number of electrons can rotate in stable motion, i. e., steadily in one ring. If more electrons are allowed the system breaks up into other rings; this can go on to a somewhat limited extent, and atoms having within them circulating systems of a given order might be expected to behave themselves more or less similarily; in other words, to have group characteristics and thus to deserve certain places in the Periodic Table.

Working upon this basis a very interesting arrangement of the elements has been constructed, into which, however, we have no time to enquire. The main object today is to give you an idea of the internal activity of the atom. This attempt I will conclude with the following statements: that if two million molecules of hydrogen could be arranged in a row they would occupy one millimeter (a twenty-fifth of an inch). Fifteen thousand million, million, million of them would weigh a grain; while when hydrogen is at its own freezing point each molecule makes on an average nearly eighteen million collisions per second, at each of which its speed is altered and diverted, yet on the average it has a speed of over six thousand feet per second, while it moves only about a quarter of a millionth of an inch between collisions. In one cubic inch, then, of hydrogen, under standard temperature and pressure, there must be about six million, million, million molecules; this number holding for all gases in accordance with Avogadro's Law.

### ETHER.

Sir Isaac Newton was perhaps the first to employ the term—Ether—for that medium which fills space, space which not only appears to be empty, but that which *seems* to be full, because ether must penetrate and permeate between the atoms and exist in the interspaces of every material in the universe. Newton postulated the elasticity of this ether, and in one of his "Questions" enquired whether the planets, comets, and all gross bodies could not perform their motions more freely in a medium denser than quicksilver and gold, and whose resistance was so small as to be inconsiderable.

Newton's views, as among the earliest of value, may well be contrasted with those recent expressions by Sir Oliver Lodge ("The Ether of Space." New York, Harper & Bros., 1909), who, in 1909, stated that he was able to advocate a view of the ether that makes it not only uniformly present and all prevading, but also massive and substantial beyond conception. It is turning out to be the most substantial thing, perhaps the only substantial thing, in the material universe. Compared to ether the densest matter, such as lead and gold, is a filmy, gossamer structure like a comet's tail, or a milky way, like a salt in a very minute solution. This view of Lodge's is but an extension of the views held by Clerk-Maxwell, when he wrote the article on Ether, in the ninth edition of the Encyclopedia Britannica, and who concluded it by saying—"Whatever difficulties we may have in forming a consistent idea of the constitution of the ether there can be no doubt that the inter-planetary and interstellar spaces are not empty, but are occupied by a material substance or body which is certainly the largest and probably the most uniform body of which we have any knowledge."

To us who have been brought up on a very different concept of the ether it is extremely difficult to regard it as do these most modern scientists. We have been too accustomed to judge externals through the medium of our sensations alone, unaided by our reason. It is the latter which postulates and demonstrates this concept, and we have now to adjust our sense perceptions in accordance with it. Thus regarded we better appreciate how only a small portion of vibrations transmitted are interpreted as light, and how many other kinds of vibratory activity may produce other effects, or appeal as they undoubtedly do to the peculiar sense organs of other living animal forms or, for that matter, even of vegetables. Reduced to their lowest terms all sensations become chemical.

The demands made in this effort correspond only with those made upon it. A body can only immediately act upon that which is in contact with it. Some contactual relation is absolutely necessary, and radiation is but one expression of it. As Lodge reminds us there is between the earth and the sun a gigantic, gravitative pull, equivalent to a force more than that which one million, million steel rods, each seventeen feet in diameter, could stand. Now, what mechanism can transmit so gigantic a force?

What I may say of ether here is of the briefest, and only with the view to notifying you of the demands made by modern science. The motions make figures run riot in our minds, and we become lost in the immensity of the calculations, as well as in their refinement. Lord Kelvin held that the amount of actual matter in space, compared with the volume of the space which it occupies, is infinitesimal; that is, the volume of space is infinitely greater than the total bulk of matter which it contains; otherwise the combined force of gravity would be far greater than actual observation reveals and now appears, that the densest material is of extraordinarily insignificant massiveness as compared with the unmodified ether which occupies by far the greater proportion, if not all, of its bulk, i. e., of said space.

And now we are taken still further. when we try to realize with Lodge, that matter is really a *manifestation of the ether,* and that those materials which we have regarded as most dense and solid are but similarily shaped masses of ether which in their own makeup and limits are somewhat less dense or more rarefied. Let me quote another sentence from Lodge, who says (page 103)—"For in every cubic millimeter of space we have, according to this view, a mass equivalent to what, if it were matter, we should call a thousand tons, circulating internally, every part of it, with a velocity comparable to the velocity of light, and therefore containing an amount of energy equal to that of a million horse-power station working continuously for forty million years"; or, as he says, again—"Every cubic millimeter of the universal ether of space must possess the equivalent of a thousand of tons of ordinary matter, and every part of it must be squirming internally with the velocity of light."

Does not this stagger the imagination and the mental ability of any of us? Or, again, as when he says "that the force with which the moon is held in its orbit would be great enough to tear asunder a steel rod four hundred miles thick." For those of us who were brought up on the old, and have now to accept this teaching, this staggering extravagance in figures can only make one think of the man who, having amassed a fortune, spends thousands of dollars where he used to begrudge pennies.

Let me conclude these remarks about the ether, quoting once more from Lodge—"That the ether of space is a continuous incompressible, stationary, fundamental substance; that matter is composed of modified and electrified specks or minute structures of ether, which are amenable to mechanical as well as to electrical force, and which add to the optical or electric density of the medium, and that all kinds of energy are due to an excessively fine-grained ethereal circulation, with an intrinsic kinetic energy of the order of $10^{-33}$ ergs per cubic centimeter."

(Erg—Force to move 1 gr. 1 cm.)

### References.

Light Energy—Margaret A. Cleaves, 1904. (Rebman Co., New York).

Introduction to Science—J. A. Thomson. (Henry Holt & Co., New York).

The Foundations of Science—H. Poincaré. (*Science Press,* New York). 1913.

Elementary Chemical Theory—J. M. Wadmore, 1912. (Van Nostrand, New York).

Laboratory Manual of Qualitative Analysis—1911. Walker & Mohan. (Privately printed).

Chemical Theory and Calculations—Wilson & Heilbron, 1912. (Van Nostrand, New York).

Contemporary Chemistry—E. Fournier d'Albe, 1911. (Van Nostrand, New York).

Triumphs and Wonders of Modern Chemistry—G. Martin, 1911. (Van Nostrand, New York).

The Study of Stellar Evolution—G. Hale, 1908. (University of Chicago Press, Chicago, Ill.)

The Colloidal and Crystalloidal State of Matter—P. Rohland, 1913. (Van Nostrand, New York, N. Y.)

Introduction to the Rarer Elements—P. Browning, 1912. (Wiley & Sons, New York, N. Y.)

The Elements—Sir Wm. Tilden, 1910. (Harper Bros., New York, N. Y.)

Elements and Electrons—Ramsay, 1912. (Harper Bros., New York, N. Y.)

The Pressure of Light—J. H. Poynting, 1910. (E. S. Gorham, New York, N. Y.)

Beyond the Atom—J. Cox, 1913. (Putnam's Sons, New York, N. Y.)

The Ether of Space—Sir Oliver Lodge, 1909. (Harper Bros., New York, N. Y.)

An Introduction to the Science of Radio-Activity—Raffety, 1909. (Longmans, Green & Co., New York, N. Y.)

The Radioactive Substances—Makower, 1908. (Appleton & Co., New York).

Radioactivity—Rutherford, 1904. (Macmillian Co., New York, N. Y.)

Radioactivity—Soddy, 1904. (Van Nostrand Co., New York, N. Y.)

The Interpretation of Radium—Soddy, 1912. (Putnam's Sons, New York).

Uranium, Radium and Vanadium—Moore & Kithil, 1913. (Government Printing Office, Washington, D. C.)

Radium Therapy—Wickham & Degrais, 1910. (Funk & Wagnalls Co.)

Radium Explained—Hampson, 1905. (Dodd, Mead & Co., New York).

Radium and Radioactivity—Cameron, 1912. (Gorham, New York, N. Y.)

Radium and Radioactive Substances—Baskerville, 1905. (Williams, Brown & Earle, Philadelphia, Pa.)

---

Identical Anomalies in Twins With Hereditary Syphilis. Lefour and Balard, *Jour. de Med. de Bordeaux,* August 31, 1913, report twins which died soon after birth. Both had widely open sutures and fontanelles, the larger one actual hydrocephalous; both had right talipes equino-varus and lumbar spina bifida, the tumor being about the size of a large mandarin orange. Both were males, one weighing 3,220 grams, the other 2,270.

---

Congenital Torsion of the Penis. Ayguesparsse, *Jour. de Med. de Bordeaux,* August 31, 1913, alludes to 11 personal observations and 8 from the literature, presented to the Bordeaux Medical Society, January 17, 1910 by Rocher. Rocher found that the torsion affected the entire organ, not merely the urethra and that it was always in the same direction, corresponding to the VI-XII half of the clock, XI representing a complete half turn, which may be exaggerated so as to point to I. Ayguesparsse reports a case in which the turn was between IV and V and accompanied with hypospadias.

